# Proteomic Profiling of Serum Exosomes From Patients With Metastatic Gastric Cancer

**DOI:** 10.3389/fonc.2020.01113

**Published:** 2020-07-10

**Authors:** Xiao-Qing Ding, Zhe-Ying Wang, Di Xia, Rui-Xian Wang, Xiao-Rong Pan, Jian-Hua Tong

**Affiliations:** Faculty of Medical Laboratory Science and Central Laboratory, Ruijin Hospital, Shanghai Jiao Tong University School of Medicine, Shanghai, China

**Keywords:** metastatic gastric cancer, exosomes, proteomics, proteasome, PSMA3, PSMA6

## Abstract

**Background:** Clinical management of metastatic gastric cancer (mGC) remains a major challenge due to a lack of specific biomarkers and effective therapeutic targets. Recently, accumulating evidence has suggested that exosomes play an essential role in cancer metastasis and can be an excellent reservoir of novel biomarkers and candidate therapeutic targets for cancer. Therefore, in this study, we aimed to reveal the proteomic profile of mGC-derived exosomes.

**Methods:** Exosomes were isolated from pooled serum samples of 20 mGC patients and 40 healthy controls (HC) by ultracentrifugation. Next, quantitative proteomic analyses were applied to analyze the protein profiles of the exosomes, and bioinformatic analyses were conducted on the proteomic data. Finally, the expression of exosomal protein candidates was selectively validated in individual subjects by western blot analysis.

**Results:** We isolated exosomes from serum samples. The size of the serum derived exosomes ranged from 30 to 150 nm in diameter. The exosomal markers CD9 and CD81 were observed in the serum exosomes. However, the exosomal negative marker calnexin, an endoplasmic reticulum protein, was not detected in exosomes. Overall, 443 exosomal proteins, including 110 differentially expressed proteins (DEPs) were identified by quantitative proteomics analyses. The bioinformatics analyses indicated that the upregulated proteins were enriched in the process of protein metabolic, whereas the downregulated proteins were largely involved in cell-cell adhesion organization. Surprisingly, 10 highly vital proteins (UBA52, PSMA1, PSMA5, PSMB6, PSMA7, PSMA4, PSMA3, PSMB1, PSMA6, and FGA) were filtered from DEPs, most of which are proteasome subunits. Moreover, the validation data confirmed that PSMA3 and PSMA6 were explicitly enriched in the serum derived exosomes from patients with mGC.

**Conclusion:** The present study provided a comprehensive description of the serum exosome proteome of mGC patients, which could be an excellent resource for further studies of mGC.

## Introduction

Gastric cancer (GC), a common digestive tract cancer, is currently the fifth most prevalent malignancy and the third leading cause of cancer associated fatalities globally (1). It has also become the second deadliest malignancy in China (2). Gastrectomy could be a potentially curative therapy for GC patients without metastasis. However, more than 35% of gastric cancer patients are diagnosed initially with metastatic gastric cancer (mGC) (3). The outcome of these mGC patients is abysmal, with approximately 4 months of median survival time (4). Even when treated with cytotoxic chemotherapy combinations or multimodal treatments, the outcome of patients with mGC remains unsatisfactory (3, 5, 6). Therefore, it is urgent to explore novel biomarkers and effective therapeutic targets for better management of patients with mGC.

Exosomes are nanometer-sized (30–200 nm) membrane-enclosed extracellular vehicles (EVs) (7, 8). They originate intracellularly in endosomes and are released into the extracellular environment when multivesicular bodies (MVBs) fused with plasma membranes (9–11). In 1980, exosomes were thought to represent non-specific waste that resulted from the maturation of reticulocytes into erythrocytes (12, 13). Currently, these small EVs are widely suggested to serve as essential mediators of different physiological and pathological processes (14, 15). Exosomes harbor a diverse of functional molecules (including proteins, nuclear acids, and lipids) derived from their originating cells, and they have been found in various body fluids, such as blood, urine, and saliva (16–20). Due to the protection of the lipid bilayer, exosomes are relatively stable. The proteins and other molecules enclosed within these small vesicles are protected from degradation (21, 22). These characteristics make exosomes great sources of non-invasive biomarkers or therapeutic targets for cancers.

Cancer exosomes, which are distal mediators of intercellular communication, have been described as playing key roles in cancer metastasis (23, 24). Several studies have shown that cancer derived exosomes can promote metastasis by remodeling the tumor microenvironment and altering the extracellular matrix to facilitate the spread and colonization of metastatic cancer cells (25–28). In particular, Deng and colleagues demonstrated that exosomes from gastric cancer could promote peritoneal metastasis by destroying the mesothelial barrier (29). In addition, some exosomal proteins have been found to be involved in GC metastasis. For example, TGF-β1 was found abundant in exosomes from GC patients, and the expression level of exosomal TGF-β1 was correlated with lymph node metastasis (30). Besides, exosomal EGFR secreted from the primary gastric tumor could be delivered to liver stromal cells, and it could increase the expression of HGF to promote liver metastasis (31). These findings suggested that exosomal proteins may play an essential role in the metastasis of GC. Therefore, it is necessary to have a deep understanding of the protein profiles of mGC-derived exosomes.

As mentioned above, accumulating data have indicated that several exosomal proteins are involved in the metastasis of gastric cancer. We conceive that serum exosomal proteins could be promising resources for further screening of potential biomarkers and therapeutic targets in mGC. However, the exploration of the proteomic profile of serum-derived exosomes from mGC patients remains absent. In this study, we intended to reveal the global proteomic profile of mGC-derived exosomes by quantitative proteomic analysis and then explore the potential biological functions of these exosomal proteins by using bioinformatic analyses. We expect that our results could provide unique resources for further studies of mGC.

## Materials and Methods

### Patients and Serum Samples

The patients in our study were limited to newly diagnosed with GC at Ruijin Hospital (Shanghai, China). Serum samples from 20 mGC patients and 40 healthy controls were used for proteomics analysis in the discovery phase. Then, serum samples from 24 GC patients and 13 healthy individuals were used for validation. The detailed clinical characteristics of all the participants are provided in Table S1. Whole blood samples were obtained in vacuum pro-coagulation tubes (BD, USA) from GC patients and healthy individuals before treatment. The blood samples without lipidemia or hemolysis were centrifuged at 2,000 × *g* for 15 min (4°C) for the preparation of serum samples. All serum samples were aliquoted and stored at −80°C for subsequent exosome isolation. The Ethics Committee of Ruijin Hospital approved this study.

### Exosome Isolation

Exosomes were isolated from serum samples by ultracentrifugation (UC) or commercial kits. For proteomics analysis, the serum samples obtained from patients with mGC and healthy individuals were pooled together, respectively. These two groups of pooled serum samples were used for exosome isolation by UC using a previously published protocol with minor modifications (32). Briefly, to decrease the viscosity, 10 ml of pooled serum was diluted five times with phosphate-buffered saline (PBS). The diluted serum samples were centrifuged at 500 × *g* for 5 min and at 2,000 × *g* for 10 min (4°C) to eliminate cells and cell debris contamination. The supernatant was further centrifuged at 10,000 × *g* for 30 min (4°C). Then, the supernatant was filtered with a 0.45 μm syringe filter (Millipore, USA) and ultracentrifuged at 100,000 × *g* for 2 h (4°C) (Optima L-100XP, 70.1 Ti rotor, Beckman Coulter) to pellet the exosomes. Then, the exosomes were resuspended in PBS and pelleted again by ultracentrifugation at 100,000 × *g* for 80 min (4°C). This cleanup step was repeated one additional time. The final exosomes pellet was resuspended in 200 μl of 0.22 μm-filtered PBS for subsequent analysis.

For validation, exosomes were isolated from the serum of individual subjects using the exoEasy Maxi Kit (Qiagen). According to the manufacturer's instructions, serum was centrifuged at 16,000 × *g* for 10 min (4°C) to remove large vesicles. Then, 2 ml of XBP buffer was added to 2 ml of precleared serum. The mixture was added to the exoEasy spin column, which was centrifuged at 500 ×*g* for 1 min (4°C). The flow-through was discarded, and 4 ml XWP buffer was added and centrifuged at 5,000 × *g* for 5 min (4°C) to wash the exosomes. The spin-column was then transferred to a new collection tube, and 400 μL of Buffer XE was added to dissolve the exosomes, followed by centrifugation at 5,000 × *g* for 5 min (4°C) to collect the eluate. The purified exosomes were then either used immediately or stored at −80°C.

### Transmission Electron Microscope (TEM)

The exosome morphology was observed by a transmission electron microscope with negative staining. Briefly, 10 μL of purified exosomes were loaded onto a copper grid for 1 min, and the excess exosomal suspension was carefully removed with filter paper. The absorbed exosomes were stained with 2% uranyl acetate for 1 min, and the excess fluid was removed with filter paper. Finally, the images of exosomes were captured under a transmission electron microscope (Tecnai G2 Spirit, FEI, Czech Republic) after the grids were dried.

### Nanoparticle Tracking Analysis (NTA)

The size distribution of the serum exosomes was analyzed using a NanoSight NS300 instrument (Malvern, UK) equipped with nanoparticle particle tracking software (Version NTA 3.2). According to the manufacturer's recommendation, the samples were illuminated by the laser (Blue 488), and the movement of nanoparticles due to Brownian motion was recorded for 60 s at a mean frame rate of 20 frames per second. Each process was repeated three times.

### Western Blot Analysis (WB)

For western blot analysis, exosomes were lysed in RIPA buffer (#9806, Cell Signaling Technology) with Protease Inhibitor Mixture (Roche Diagnostics, Germany) and quantified using a BCA protein assay reagent kit (Pierce, USA). The protein samples were denatured at 95°C for 10 min in 5 × Laemmli buffer. Then, 20 μg of protein was separated by 10% or 12% SDS-PAGE and transferred onto Hybond-C Extra membranes (Amersham Biosciences, Piscataway, NJ). After blocking with 5% skim milk, the membranes were incubated with primary antibodies, including anti-CD9 (System Biosciences, EXOAB-CD9A-1, 1:10,000), anti-CD81 (Proteintech, 18250-1-AP, 1:1000), anti-calnexin (Proteintech, 10427-2-AP, 1:5000), anti-PSMA3 (Santa Cruz Biotechnology, sc-166205, 1:1000), and anti-PSMA6 (Santa Cruz Biotechnology, sc-271187, 1:1000) overnight. Then, the membranes were incubated with a secondary antibody (Cell Signaling Technology, 1:5000) for 1 h at room temperature. Each step was followed by washing in 1 × TBS-T for 10 min 3 times each. The immunoreactive blots were visualized using a chemiluminescence kit (Millipore, Billerica, MA) and imaged with a Tanon 5200 Multi-imaging system (Tanon, Shanghai, China). Densitometric analysis was performed using the western blot analysis images using Gel-Pro Analyzer software (Media Cybernetics, United States).

## Proteomic Analysis

### Exosomal Protein Lysis and Digestion

Exosomes from the two sample groups (mGC and HC) were solubilized in lysis buffer (8 M urea and 1% protease inhibitor cocktail). Then, the protein concentration was determined with the BCA kit according to the manufacturer's instructions. Equal amounts of protein (100 μg) from the two groups were reduced with dithiothreitol and alkylated with iodoacetamide. Then, the protein samples were diluted until the urea concentration was <2 M by adding 100 mM triethylammonium bicarbonate buffer (TEAB). Subsequently, trypsin was added to the sample at a 1:50 (w/w) trypsin-to-protein ratio for the first overnight digestion and a 1:100 (w/w) trypsin-to-protein ratio for the second 4 h digestion.

### Peptide Fractionation

The peptides were fractionated by high pH reverse-phase High-Performance Liquid Chromatography (HPLC) with an Agilent 300 Extend C18 column (5 μm diameter, 4.6 mm inner diameter, and 250 mm length). Briefly, the peptides were first separated with a gradient of 8–32% acetonitrile (pH 9.0) over 60 min into 60 fractions. Then, the peptides were combined into four fractions and dried by vacuum centrifugation.

### Liquid Chromatography Coupled to Tandem Mass Spectrometry (LC-MS/MS)

The peptides were dissolved in 0.1% (v/v) formic acid and then separated by an EASY-nLC 1000 ultra-high-performance liquid system. Mobile phase A consisted of 0.1% formic acid and 2% acetonitrile, and mobile phase B consisted of 0.1% formic acid and 90% acetonitrile. After separation, the peptides were injected into the NSI source for ionization and then analyzed by an Orbitrap Fusion MS (Thermo Scientific, USA). The electrospray voltage was set at 2.0 kV. The scanning range for primary mass spectrometry was set to 350–1,550 m/z, and the scanning resolution was set to 60,000; the fixed starting point of the scanning range for secondary mass spectrometry was 100 m/z, and the scanning resolution for secondary mass spectrometry was set to 15,000. Data acquisition mode used a data-dependent scanning (DDA) program.

### Data Processing

The raw MS/MS data were processed using the MaxQuant search engine (v.1.5.2.8 http://www.maxquant.org/). The tandem mass spectra were queried against the Human UniProt/SwissProt database combined with the reverse decoy database. The Trypsin/P was specified as the cleavage enzyme, and up to two missed cleavages were allowed. The mass tolerance value for precursor ions was set to 20 ppm for the first search and 5 ppm for the main search. The mass tolerance value for the fragment ions was set to 0.02 Da. The FDR was set to <1%. Proteins were quantified using label-free quantification, and the relative protein abundances are presented as the mGC/HC ratios. The differential expression threshold was set to a 2-fold change.

### Bioinformatics Analysis

The subcellular localization of all identified proteins was predicted by wolfpsort (v.0.2 http://www.genscript.com/psort/wolf_psort.html), which is a protein subcellular localization prediction tool.

For the Gene Ontology (GO) enrichment analysis, proteins were classified by GO annotation into three categories, biological process, cellular compartment, and molecular function, according to the UniProt-GOA database (http://www.ebi.ac.uk/GOA/). Fisher exact test was performed to compare the enrichment of the differentially expressed proteins to that of all identified proteins. A corrected *p* < 0.05 was considered significant.

A famous public pathway database, Kyoto Encyclopedia of Genes and Genomes (KEGG) (http://www.genome.jp/kegg/), was used to identify the enriched pathways by Fisher's exact test to compare the enrichment of the differentially expressed proteins to that of all identified proteins. A corrected *p* < 0.05 was considered significant.

The STRING database (https://stringdb.org/; v.10.5) was used for protein-protein interaction network (PPI) analysis (33). Only interactions between proteins included in the data search set were selected, thereby excluding external candidates. The interaction network generated by using STRING was visualized in Cytoscape (http://www.cytoscape.org/, v.3.7.1) (34, 35). The cytoHubba plug-in of Cytoscape software was used to select the essential hub proteins among the DEPs, which provided the analysis results determined according to the maximal clique centrality (MCC) method (36).

### Statistical Analysis

The exosomal PSMA3 and PSMA6 levels in GC patients and healthy controls were compared by the Mann-Whitney U-test using GraphPad Prism 7 software, respectively. A two-sided *p* < 0.05 was defined as statistically significant.

## Results

### General Experiment Design

**Figure 1:**
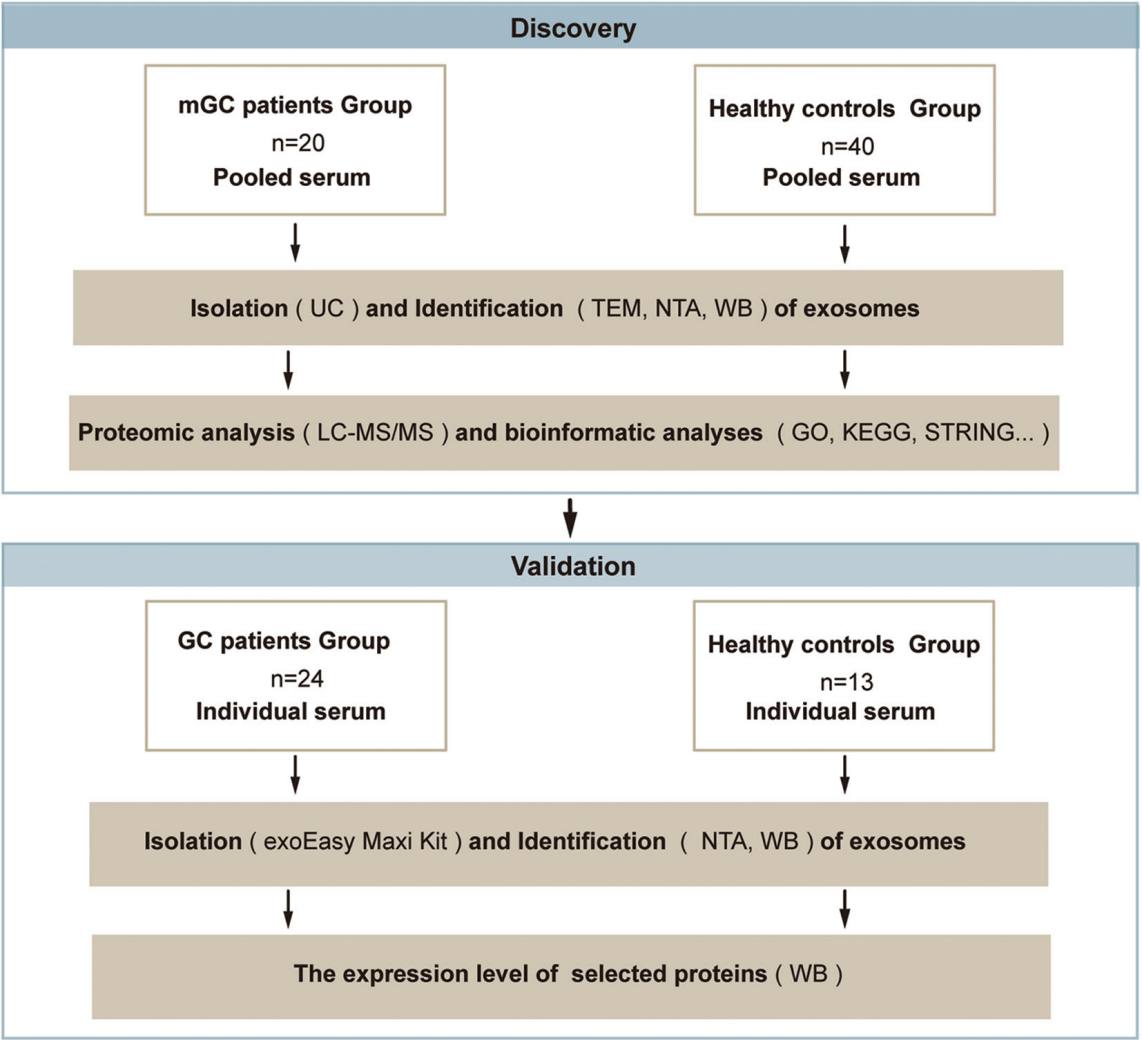
Schematic overview of the study based on the proteomic analysis. Discovery phase: to discover the exosomal proteins differentially expressed between mGC patients and healthy controls. Validation phase: to validate the selected differentially expressed candidate protein. Abbreviations: mGC, metastatic gastric cancer; GC, gastric cancer.

To obtain a comprehensive knowledge of proteins in the serum exosomes from mGC patients, we designed a strategy based on proteomic analysis, as presented in Figure 1. In the discovery phase, serum samples from patients diagnosed with mGC (*n* = 20, stage IV) and healthy controls (*n* = 40) were collected and pooled (Table S1). We isolated exosomes from the pooled serum samples by UC and characterized by TEM, NTA, and WB. Next, proteomic analysis based on LC-MS/MS was performed to identify the protein profiles of the exosome samples and determine the exosomal proteins differentially expressed between mGC patients and healthy controls. Then bioinformatics analyses, including GO function enrichment analysis, KEGG pathway enrichment analysis, and PPI analysis, were performed on differentially expressed proteins. After the bioinformatics analyses, we selected candidate proteins for further validation. In the validation phase, exosomes were isolated from individual serum samples of 24 GC (stage I–IV) patients and 13 healthy individuals (Table S1) using the exoEasy Maxi Kit and identified by NTA and WB. Subsequently, the expression levels of the selected proteins in these exosome samples were compared by WB.

### Isolation and Characterization of Exosomes

**Figure 2:**
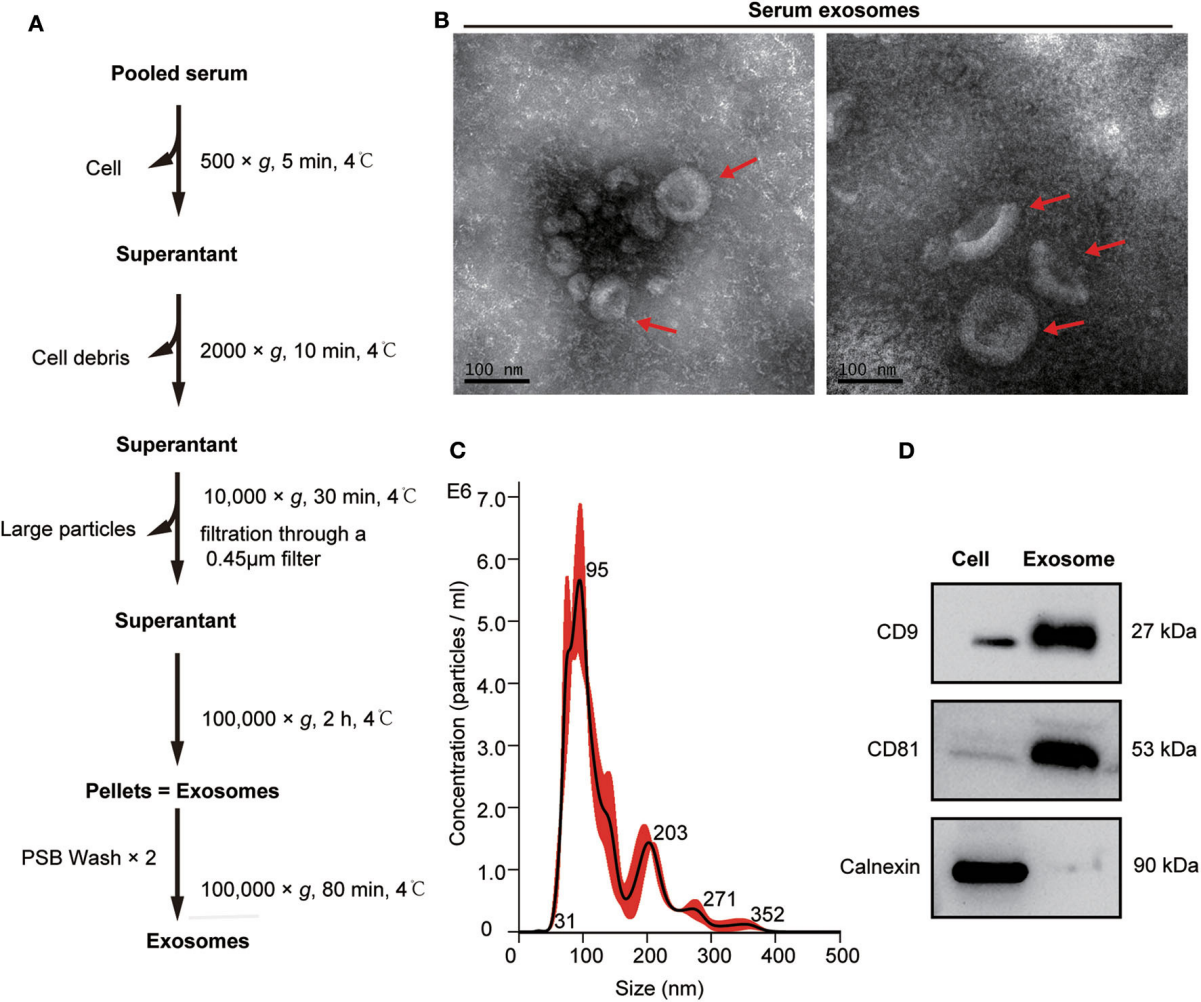
Isolation and characterization of serum exosomes. **(A)** Flow chart of exosome isolation from serum samples using ultracentrifugation (UC). **(B)** Transmission electron microscope (TEM) images of exosomes isolated from serum shown at magnification of 110,000 ×. The bar represents 100 nm. Red arrows indicate typical cup-shaped exosomes. **(C)** The size distribution of serum exosomes determined by nanoparticle tracking analysis (NTA). **(D)** Western blot analysis of exosome markers (CD9 and CD81) and negative markers (calnexin) in equivalent amounts of protein (20 μg) from serum exosomes and 293 T cell lysates(as a control).

Exosomes from the pooled serum samples of healthy controls and patients with mGC were isolated by UC, as described in Figure 2A. The following TEM, NTA, and WB analyses were performed on exosomes isolated from serum samples. TEM analysis revealed cup-shaped vesicles with a size range of 50–150 nm in diameter (Figure 2B), similar to previously reported descriptions of exosomes (14, 37). NTA showed that the mean size of purified exosomes was 87.0 ± 6.5 nm, and the primary peak size was 95 nm (Figure 2C). Moreover, WB analysis revealed that exosomal marker proteins (CD9 and CD81) were significantly expressed in the exosome samples. In contrast, calnexin, which generally represents contamination by intracellular proteins, was absent (Figure 2D). Altogether, these results demonstrated that we successfully isolated exosomes from clinical serum samples.

### Comprehensive Proteomic Analysis of Exosomes

**Figure 3:**
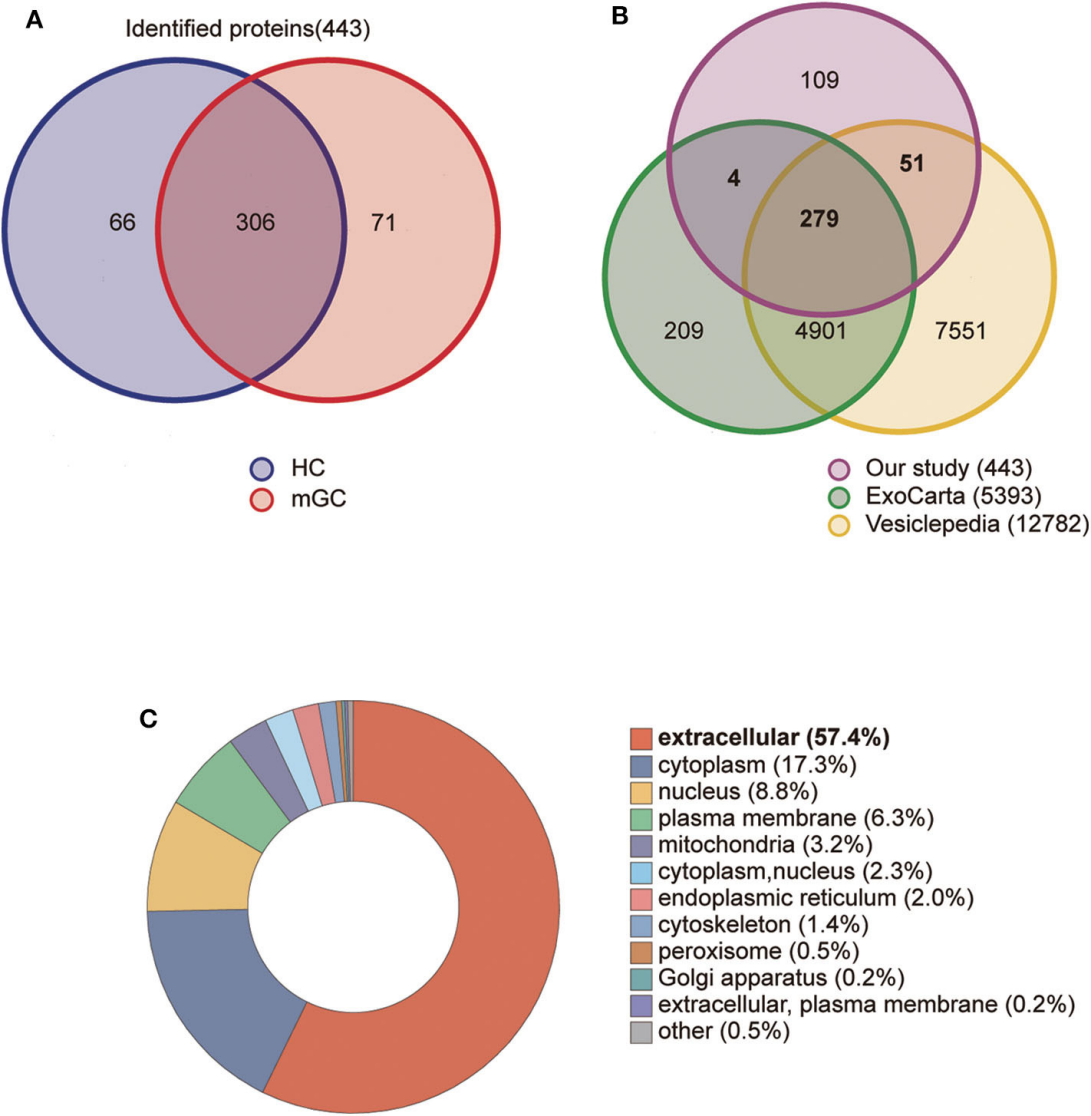
Proteomic analysis of serum derived exosomes. **(A)** The Venn diagram displays the distribution of exosomal proteins between mGC patients and healthy controls (HC). **(B)** The overlap of proteins identified in the present study with those in the Exocarta and Vesiclepedia databases (ExoCarta Version 5, Release date: 29 July 2015; Vesiclepedia Version 4.1, Release date: 15 August 2018). **(C)** The subcellular location of the total identified proteins predicted by wolfpsort on-line tool.

In the present study, a total of 443 unique proteins were identified in serum exosomes by label-free quantitative proteomic analysis. Among them, 377 and 372 proteins were identified in mGC patients and healthy controls, respectively (Figure 3A). We compared all the proteins identified in our study to those known vesicular proteins in the Exocarta (38, 39) and Vesiclepedia databases (40, 41). Among the 443 identified proteins, 334 (75.4%) proteins were found in previously published data (Figure 3B, Table S2), including the common exosomal markers CD9, CD81, flotillin and syntenin. In addition, 109 probable exosomal proteins were also newly identified, which are not present in the Exocarta and Vesiclepedia databases (Figure 3B, Table S2). These data confirmed that the exosomes from our preparations contained abundant exosomal proteins. Additionally, we predicted the subcellular localization of all the identified proteins by wolfpsort. Most of the identified proteins (57.4%) were located in the extracellular region (Figure 3C). For further exploration, 110 differentially expressed proteins (DEPs) were screened from the results based on the differential expression threshold (fold change > 2 times). Among the 110 DEPs, 64 proteins were upregulated (Table 1), while 46 proteins were downregulated (Table 2) in serum exosomes samples isolated from mGC patients relative to those isolated from healthy controls. Detailed information on the DEPs can be found in Table S3.

**Table 1:** The upregulated DEPs between mGC patients group and healthy controls group.

**No**.	**Protein accession**	**Gene name**	**mGC/HC Ratio**	**No**.	**Protein accession**	**Gene name**	**mGC/HC Ratio**
1	O14818	PSMA7	2.08	33	P04180	LCAT	Inf+
2	P00738	HP	2.13	34	P04211	IGLV7-43	Inf+
3	Q9Y251	HPSE	2.13	35	P08311	CTSG	Inf+
4	P02750	LRG1	2.35	36	P08709	F7	Inf+
5	P59666	DEFA3	2.38	37	P08758	ANXA5	Inf+
6	P30626	SRI	2.45	38	P0DJI8	SAA1	Inf+
7	P02765	AHSG	2.54	39	P11678	EPX	Inf+
8	A0A075B6I0	IGLV8-61	2.60	40	P20618	PSMB1	Inf+
9	P05164	MPO	2.61	41	P25786	PSMA1	Inf+
10	P02786	TFRC	2.70	42	P25788	PSMA3	Inf+
11	P68431	HIST1H3A	2.79	43	P25789	PSMA4	Inf+
12	P02671	FGA	2.84	44	P28066	PSMA5	Inf+
13	Q99880	HIST1H2BL	2.89	45	P28072	PSMB6	Inf+
14	P20742	PZP	2.97	46	P35443	THBS4	Inf+
15	P06681	C2	3.14	47	P49913	CAMP	Inf+
16	P22891	PROZ	3.30	48	P54108	CRISP3	Inf+
17	Q99878	HIST1H2AJ	3.69	49	P55058	PLTP	Inf+
18	P01704	IGLV2-14	4.07	50	P55072	VCP	Inf+
19	P00742	F10	4.42	51	P60900	PSMA6	Inf+
20	P11597	CETP	4.65	52	P62987	UBA52	Inf+
21	P02679	FGG	4.91	53	P80511	S100A12	Inf+
22	P04070	PROC	5.32	54	Q08554	DSC1	Inf+
23	P62805	HIST1H4A	5.33	55	Q08830	FGL1	Inf+
24	P02741	CRP	7.20	56	Q13790	APOF	Inf+
25	P18428	LBP	13.39	57	Q71DI3	HIST2H3A	Inf+
26	A0A087WW87	IGKV2-40	Inf+	58	Q8NEZ4	KMT2C	Inf+
27	A0A0C4DH33	IGHV1-24	Inf+	59	Q8WUJ3	CEMIP	Inf+
28	O00560	SDCBP	Inf+	60	Q8WXI7	MUC16	Inf+
29	O00602	FCN1	Inf+	61	Q9HC84	MUC5B	Inf+
30	O95810	CAVIN2	Inf+	62	Q9NZP8	C1RL	Inf+
31	P01721	IGLV6-57	Inf+	63	Q9NZT1	CALML5	Inf+
32	P02042	HBD	Inf+	64	Q9Y2I7	PIKFYVE	Inf+

*mGC, metastatic gastric cancer; HC, healthy controls; DEPs, differentially expressed proteins; Inf+, infinity*.

**Table 2:** The downregulated DEPs between mGC patients group and healthy controls group.

**No**.	**Protein accession**	**Gene name**	**mGC/HC Ratio**	**No**.	**Protein accession**	**Gene name**	**mGC/HC Ratio**
1	P07437	TUBB	0.21	24	P01763	IGHV3-48	0.49
2	P18206	VCL	0.23	25	A0A075B6J9	IGLV2-18	0.49
3	Q9Y490	TLN1	0.28	26	A0A075B6I1	IGLV4-60	Inf-
4	P21333	FLNA	0.31	27	A0A075B6K6	IGLV4-3	Inf-
5	P12814	ACTN1	0.33	28	A0A087WSX0	IGLV5-45	Inf-
6	P02792	FTL	0.33	29	A0A087WSY4	IGHV4-30-2	Inf-
7	P02794	FTH1	0.33	30	A0A0B4J1U3	IGLV1-36	Inf-
8	Q13201	MMRN1	0.34	31	O15511	ARPC5	Inf-
9	A0A0A0MRZ8	IGKV3D-11	0.36	32	O75083	WDR1	Inf-
10	Q86UX7	FERMT3	0.37	33	P01715	IGLV3-1	Inf-
11	P27918	CFP	0.39	34	P01718	IGLV3-27	Inf-
12	P03951	F11	0.39	35	P05556	ITGB1	Inf-
13	P01764	IGHV3-23	0.40	36	P07195	LDHB	Inf-
14	P12259	F5	0.40	37	P0DP02	IGHV3-30-3	Inf-
15	P60709	ACTB	0.40	38	P15814	IGLL1	Inf-
16	P04430	IGKV1-16	0.42	39	P17936	IGFBP3	Inf-
17	P68133	ACTA1	0.42	40	P23229	ITGA6	Inf-
18	A2NJV5	IGKV2-29	0.46	41	P43251	BTD	Inf-
19	P01782	IGHV3-9	0.46	42	P68366	TUBA4A	Inf-
20	Q15485	FCN2	0.47	43	P69891	HBG1	Inf-
21	P05106	ITGB3	0.47	44	Q01518	CAP1	Inf-
22	P01706	IGLV2-11	0.48	45	Q9BQE3	TUBA1C	Inf-
23	P13224	GP1BB	0.48	46	Q9C0H2	TTYH3	Inf-

*mGC, metastatic gastric cancer; HC, healthy controls; DEPs, differentially expressed proteins; Inf-, infinitesimal*.

### Functional Enrichment (GO and KEGG) Analyses of DEPs

**Figure 4:**
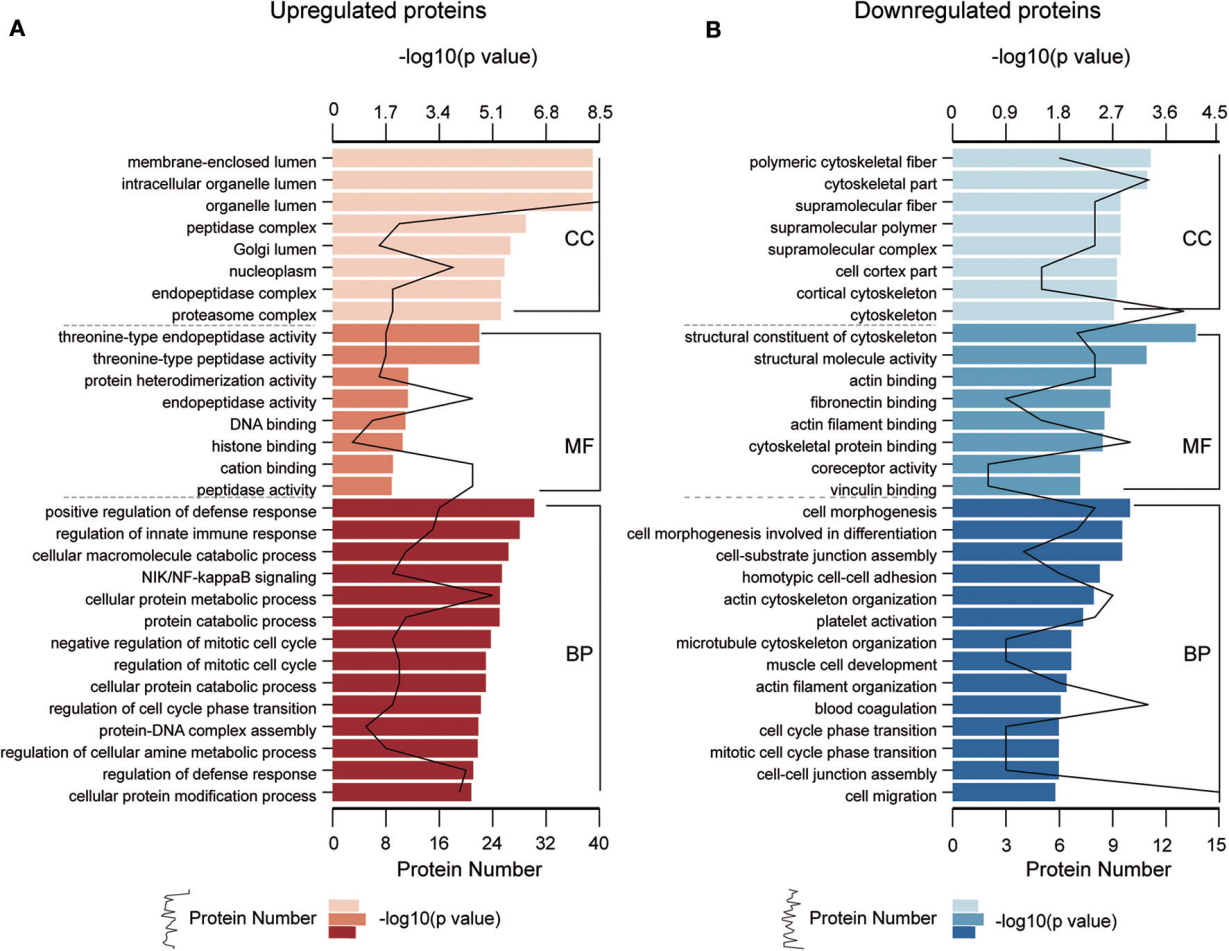
GO enrichment analyses of differentially expressed proteins (DEPs). GO enrichment analysis of upregulated DEPs **(A)** and downregulated DEPs **(B)** in the cellular component (CC), molecular function (MF), and biological process (BP) categories. All significantly enriched GO terms (*P* < 0.05) involving DEPs are displayed. The specific proteins are listed in Table S4.

**Figure 5:**
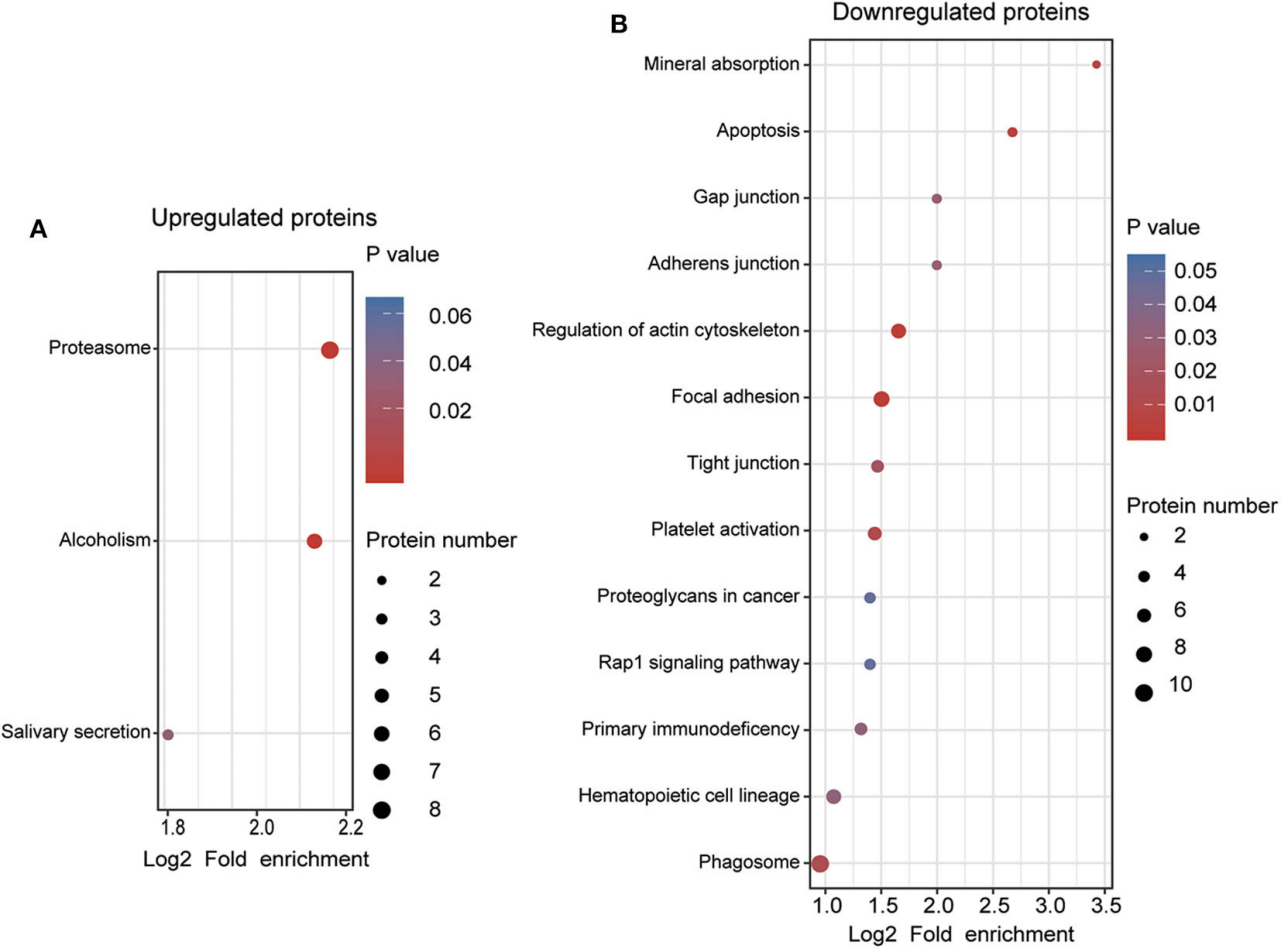
KEGG pathway enrichment analyses of differentially expressed proteins (DEPs). **(A)** KEGG pathway enrichment analysis of upregulated DEPs. **(B)** KEGG pathway enrichment analysis of downregulated DEPs. All significantly enriched KEGG pathway terms (*P* < 0.05) involving DEPs are displayed. The specific proteins are listed in Table S5.

To understand the functional significance of the DEPs, GO and KEGG pathway enrichment analyses were conducted on all 110 DEPs by using bioinformatics tools. Fisher's exact test was performed to assess the enrichment levels of DEPs. The DEPs GO enrichment analysis results were classified into three main sections: cellular component (CC), molecular function (MF), and biological process (BP). As shown in Figure 4 (Table S4), for the CC section, upregulated proteins were significantly enriched in the membrane-enclosed lumen, while the downregulated proteins were primarily enriched in polymeric cytoskeletal fibers. For the MF section, upregulated proteins were largely enriched in threonine-type endopeptidase activity; downregulated proteins were largely enriched in the structural constituent of the cytoskeleton. For the BP section, the upregulated proteins were significantly involved in the positive regulation of defense response, regulation of innate immune response, and cellular macromolecule catabolic process, while the downregulated proteins were mainly involved in cell morphogenesis, cell-substrate junction assembly, and homotypic cell-cell adhesion. Based on the KEGG pathway analyses, the DEPs were totally enriched in 16 significant KEGG pathways (*P* < 0.05). The upregulated proteins were significantly involved in the proteasome, alcoholism, and salivary secretion pathways (Figure 5A). In contrast, the downregulated proteins were significantly involved in the regulation of the actin cytoskeleton, focal adhesion, and apoptosis (Figure 5B). The detailed results of KEGG pathway analyses are shown in Table S5. Overall, through functional enrichment analyses, we found that the majority of the upregulated proteins were involved in the protein metabolic process, while downregulated proteins were primarily involved in cell-cell adhesion organization.

### PPI Analysis of DEPs and Identification of Key Proteins

Proteins usually interact with other proteins. To further explore the potential interactions among the 110 DEPs, a PPI network was developed using the STRING database and displayed with Cytoscape 3.7.1. In total, there are 73 nodes (66.4% of all 110 DEPs) and 231 edges (protein-protein associations) in this PPI network, including 47 upregulated and 26 downregulated proteins (Figure S1A). Detailed information about the protein-protein interactions is shown in Table S6. Subsequently, by adopting the MCC method in cytoHubba, the top 10 most vital proteins (UBA52, PSMA1, PSMA5, PSMB6, PSMA7, PSMA4, PSMA3, PSMB1, PSMA6, and FGA) with the highest scores were selected from the entire PPI network (Figure S1B). Interestingly, all ten prominent exosomal proteins were notably upregulated in mGC patients compared to healthy controls, and 8 of 10 hub proteins are involved in the proteasome pathway. These results are consistent with the results of the GO and KEGG pathway enrichment analyses of 110 DEPs, implying that the proteasome may act an important part in GC metastasis and should be further studied.

### Validation of Selected Protein Candidates Using Western Blot Analysis

**Figure 6:**
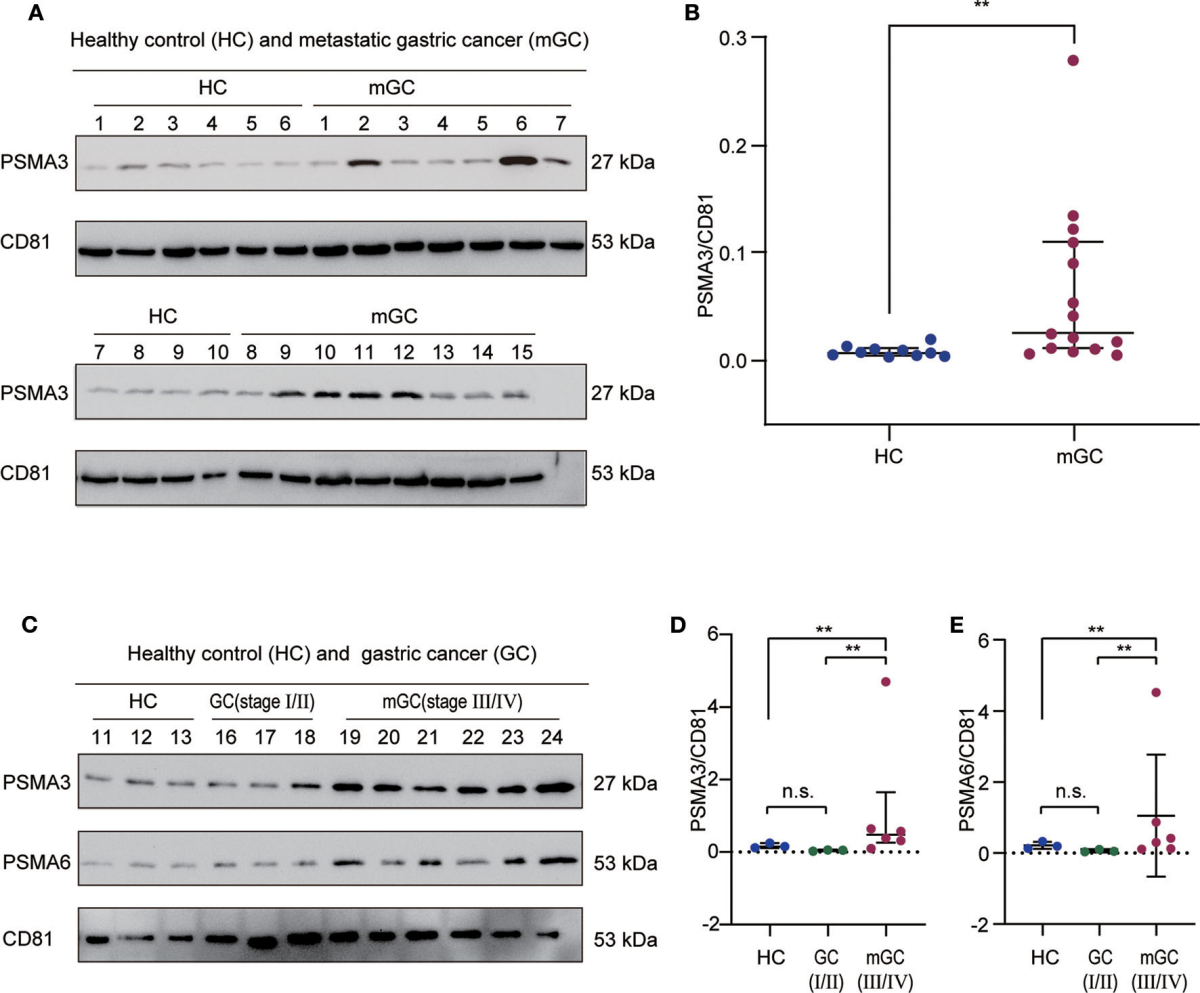
Validation of exosomal PSMA3 and PSMA6 in individual samples. **(A)** Western blot analysis of PSMA3 in exosome samples from 10 healthy controls and 15 mGC patients. CD81 was used as an internal control for equivalent amounts of protein (20 μg). The corresponding quantified data are shown in **(B)**. **(C)** Western blot analysis of PSMA3 and PSMA6 in exosome samples from 3 healthy controls and 9 GC patients at different stages. The corresponding quantified data are shown in **(D,E)**. The results are presented as the median with the 25th percentile and 75th percentile values. Mann-Whitney U-tests were used to determine significance: **p <0.01, n.s. no significance (*P* > 0.05).

According to the bioinformatics analysis and a recent research report (42), the proteasome subunit PSMA3, which is one of the hub proteins, was first chosen to validate the proteomics results. In the validation cohort (13 healthy controls and 24 GC patients), we isolated exosomes from individual serum samples utilizing the exoEasy Maxi Kit and then verified them by NTA and WB (Figure S2). The expression level of PSMA3 in serum exosome was detected by western blot analysis. CD81 was used as the internal control since it is a standard marker of exosomes. As shown in Figures 6A,B, the expression level of exosomal PSMA3 was significantly higher in mGC patients than that in healthy controls, which was consistent with our proteomics results. To further examine the expression level of exosomal PSMA3 in GC patients without metastasis, we compared the level of exosomal PSMA3 in healthy controls and GC patients at different stages. Interestingly, exosomal PSMA3 was especially upregulated in mGC patients (stage III/IV) (Figures 6C,D). No significant difference was observed between healthy controls and GC patients without metastasis (stage I/II). Additionally, we performed the same experiment on PSMA6 in healthy controls and GC patients at different stages. The result of PSMA6 is consistent with the finding of PSMA3 (Figures 6C,E).

## Discussion

Exosomes serve as a high-quality resource as a liquid biopsy tool for cancer research since they can prevent contents inside from degradation (43). Furthermore, exosomes can be used to obtain more pure samples that are devoid of proteins that are highly abundant in body fluids, which can facilitate analyses (44, 45). In recent years, proteomic analyses of exosomal proteins in blood samples have been utilized to explore possible biomarkers for cancers. For example, Chen and coworkers identified the protein profiles in exosomes from patients with CRC by proteomic analysis (46). They suggested that several exosomal proteins may be helpful for the management of diagnostics and therapeutics of CRC (46). Zhang et al. performed the proteomic profiling on plasma exosomes of ovarian cancer patients and identified a potential use for the diagnosis and prognostic prediction of patients (47). Furthermore, the proteomic profile found in serum exosomes from patients with advanced pancreatic cancer undergoing chemoradiotherapy revealed that exosomal proteins might be involved in the development of metastasis and treatment resistance (48). Based on these studies mentioned above, it is reasonable to assume that the profiles of proteins in serum exosomes derived from mGC patients may play a vital part in GC metastasis, and these exosomal proteins could be an excellent source for the exploration of serve as novel biomarkers and therapeutic targets for mGC.

The protein signatures in serum exosomes derived from mGC patients, especially those with distant metastasis, have rarely been studied before. We collected serum samples from GC patients with distant metastasis for proteomic analysis. By ultracentrifugation, we successfully isolated exosomes from serum of mGC patients and healthy controls, and these exosomes were characterized by TEM, NTA, and WB analyses to verify the high quality of the isolated exosomes. Subsequently, we conducted a comprehensive proteomic analysis of these exosomes based on LC-MS/MS. An entirety of 443 unique exosomal proteins was identified in our study. 334 (75.4%) proteins from the total exosomal proteome were matched with exosomal proteins in the ExoCarta and Vesiclepedia databases. Furthermore, 109 exosomal proteins were newly identified in our study. As expected, most of the identified exosomal proteins in our proteomic data were localized in the extracellular region. Based on a fold change > 2-fold, 110 DEPs were determined from the results. The following functional analyses (GO and KEGG pathway) and PPI network construction identified the potential biological function and mutual interactions of the DEPs, indicating that the 110 DEPs were mainly involved in the protein metabolic process and cell-cell adhesion organization. Finally, the top 10 most vital proteins (UBA52, PSMA1, PSMA5, PSMB6, PSMA7, PSMA4, PSMA3, PSMB1, PSMA6, and FGA) were selected from the PPI network, and these proteins could serve as candidate proteins for further studies. Due to a lack of effective methods for validation assays, we first selected one of the highly vital proteins for validation in another small cohort. Surprisingly, the results of WB analysis confirmed that exosomal PSMA3 and PSMA6 levels in patients with mGC were significantly higher than those in healthy controls and patients with early-stage GC, which means that proteasome subunits may be involved in the metastasis of gastric cancer through exosomes. To confirm that, further studies should be conducted to verify more candidate proteins within a larger group of patients with mGC and explore the biological function of exosomes in mGC.

In the present study, we first found that the subunits of the proteasome are significantly enriched in serum exosomes derived from mGC patients. These subunits (PSMA1, PSMA5, PSMB6, PSMA7, PSMA4, PSMA3, PSMB1, and PSMA6) belong to the 26S proteasome complex. This protease complex is a part of the ubiquitin proteasome system (UPS), which is the principal proteolytic system responsible for the functional modification and the degradation of cellular proteins (49). The proteasome has been recognized as a promising therapeutic target for inflammation and cancer (50–52). Moreover, the proteasome may be involved in the development of cancer metastasis (53, 54). Fan et al. reported that miR-127-3p could target PSMB5 to inhibit the invasion and migration of PCa cells (53). Consistent with our results, several studies found that the proteasome can be detected in exosomes (42, 55, 56). Jia et al. reported that proteasome subunits, including PSMD7, PSMD14, PSMC1, PSMD1, and PSMC2, were present in HepAD38-secreted exosomes (56). Most interestingly, Xu and coworkers recently reported that exosomal PSMA3 and PSMA3-AS1 play unique roles in multiple myeloma and may serve as hopeful prognostic predictors and therapeutic targets (42). These studies, along with our work, strongly support the hypothesis that the exosomal proteasome subunits might act as biomarkers and therapeutic targets for mGC. However, the specific molecular mechanism and potential clinical applications require further research.

In addition to the top 10 most vital proteins, we also surprisingly found other important proteins among the DEPs (Table S3), such as CEMIP (cell migration-inducing and hyaluronan-binding protein), which could play an important part in cancer metastasis. The development of cancer metastasis is a multistep and multifactorial process. Exosomes, as distal mediators of cell communication, can deliver functional cargos to adjacent or distant recipient cells. They are involved in the metastatic process, including local invasion, angiogenesis, intravasation, extravasation, and colonization of secondary organs (23, 57). CEMIP (alias KIAA1199) is involved in hyaluronan depolymerization, cell motility and migration, and extracellular matrix remodeling (58). Recently, several investigations have suggested that CEMIP is involved in the metastasis of numerous cancers, including GC. Wang et al. reported that The upregulation of CEMIP might be attributable to the lymph node metastasis of GC (59). Interestingly, an investigation has revealed that CEMIP can be enclosed in exosomes involved in cancer metastasis. David Lyden and coworkers demonstrated that exosomal CEMIP protein could be delivered to distant recipient cells (brain endothelial and microglial cells) to promote vascular remodeling and cancer metastasis (60). They also found that high levels of exosomal CEMIP were related to brain metastasis progression and patient survival (60). Altogether, their results indicated that exosomal CEMIP could be a potential therapeutic target for brain metastasis (60). Overall, these studies suggest that exosomal CEMIP may play an important role in the metastasis of GC, which is worth further exploration.

In summary, this study revealed the global protein profile of exosomes in serum from patients with mGC. We screened ten vital proteins, including eight proteasome subunits, and validated two of them (PSMA3 and PSMA6) in another cohort. These findings provide unique resources for further studies of mGC.

## Data Availability Statement

The datasets presented in this study can be found in online repositories. The names of the repository/repositories and accession number(s) can be found below: ProteomeXchange Consortium via the PRIDE partner repository with the dataset identifier PXD019387.

## Ethics Statement

The studies involving human participants were reviewed and approved by Ruijin Hospital Ethics Committee. The patients/participants provided their written informed consent to participate in this study.

## Author Contributions

X-QD, J-HT, and X-RP designed the study. X-QD, DX, and R-XW performed experiments. X-QD and X-RP analyzed data and wrote the manuscript. X-QD, X-RP, and Z-YW interpreted data and revised the manuscript. All authors contributed to the article and approved the submitted version.

## Conflict of Interest

The authors declare that the research was conducted in the absence of any commercial or financial relationships that could be construed as a potential conflict of interest.
