# Supplementary material for: Proteomic Profiling of Serum Exosomes From Patients With Metastatic Gastric Cancer
Source: Front Oncol. 2020 Jul 10;10:1113. doi: 10.3389/fonc.2020.01113 (PMC7367030; doi:10.3389/fonc.2020.01113)
Supplement: Supplementary file 2 [file Data_Sheet_2.docx]

Supplementary Material- Figure S1, Figure S2

Proteomic Profiling of Serum Exosomes from Patients with Metastatic Gastric Cancer

Xiao-Qing Ding, Zhe-Ying Wang, Di Xia, Rui-Xian Wang, Xiao-Rong Pan*, Jian-Hua Tong*

Faculty of Medical Laboratory Science and Central Laboratory, Ruijin Hospital, Shanghai Jiao Tong University School of Medicine, Shanghai, China.

* Address correspondence to the author at Ruijin Hospital, Shanghai Jiao Tong University School of Medicine, 197 Rui-jin Er Road, Shanghai 200025, China. Fax +86-21-64454908; e-mail: jh_tong@126.com or xrpan@126.com.


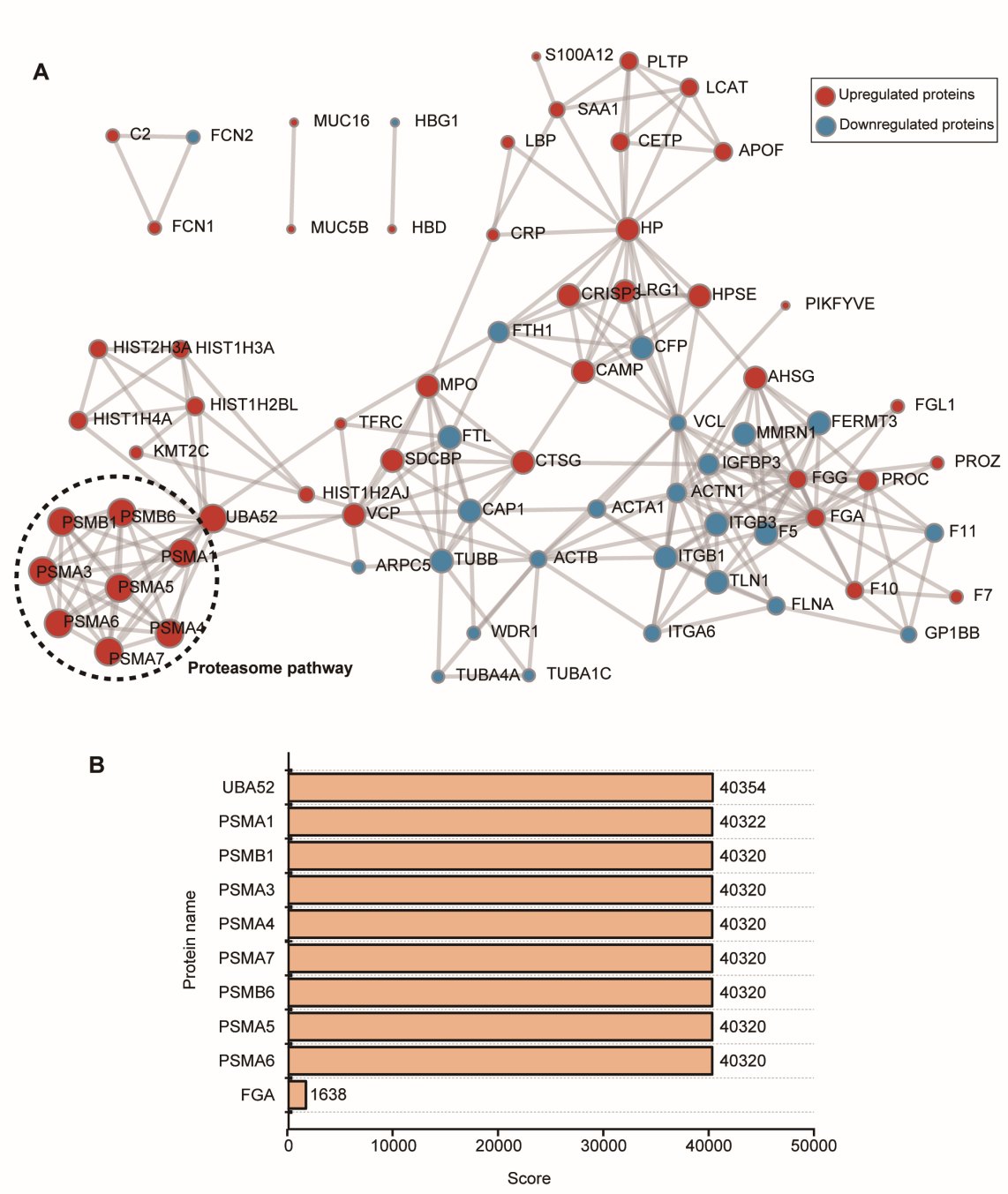


**Figure S1** Protein-protein interaction (PPI) analysis of differentially expressed proteins (DEPs). **(A)** The whole PPI network of DEPs visualized by Cytoscape software. The red nodes represent upregulated DEPs, and the blue nodes represent downregulated DEPs in mGC patients compared to the healthy controls. The edges represent the connectivity between two proteins. The size of nodes determined by degree of connectivity. The proteins in the dashed circle are the proteasome subunits involved in the proteasome pathway. **(B)** The top 10 key proteins with the highest scores were chosen from the entire PPI network by CytoHubba.


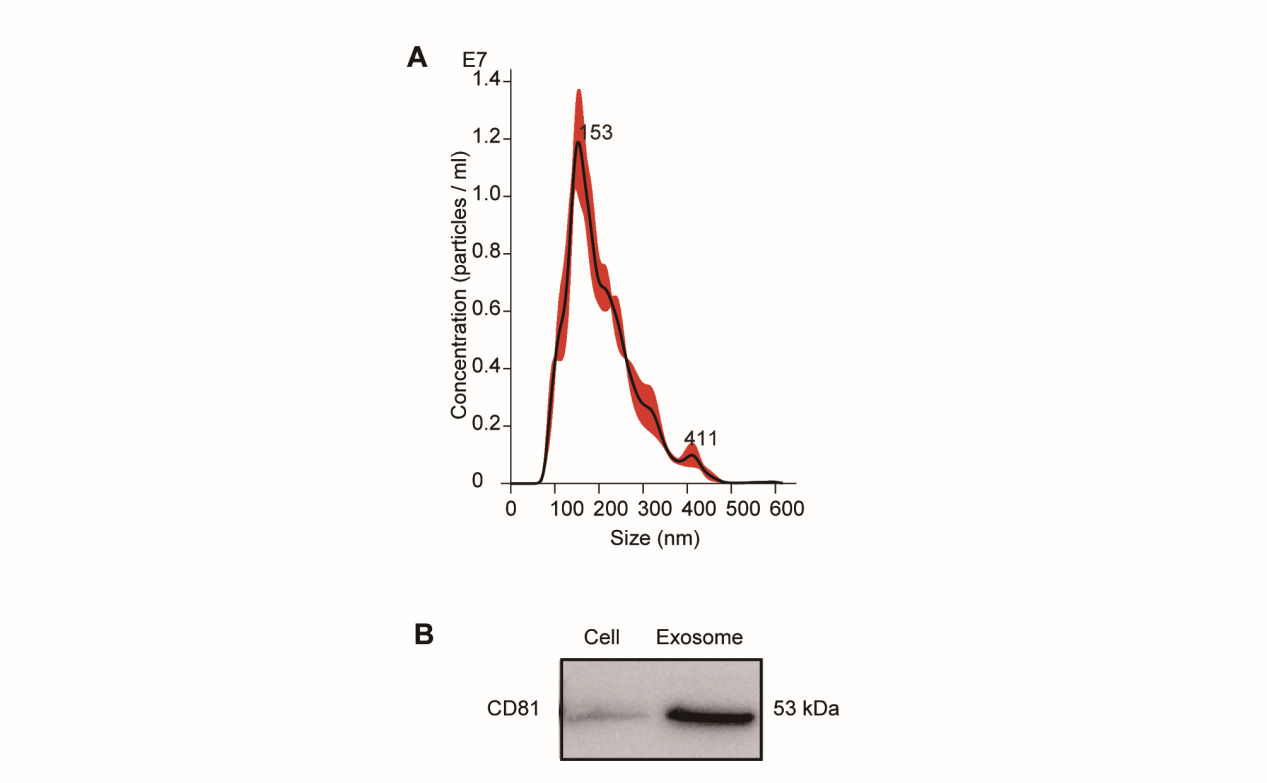


**Figure S2.** Characterization of human serum exosomes isolated by the exoEasy Maxi Kit. **(A)** Size distribution of serum exosomes determined by Nanoparticle tracking analysis (NTA). **(B)** Western blot analysis for exosome marker CD81 in equivalent amounts of proteins (20μg) from serum exosomes and 293T cell lysates.
